# Event and Comment

**Published:** 1909-03-15

**Authors:** 


### DENTAL REGISTER.

**Vol. LXIII. March 15, 1909. No. 3.**

### EVENT AND COMMENT.

DENTAL BOOKS. We have often wondered why dentistry has not produced more books. Compared with other professions, it does not average anything near the medical profession, for instance, in either its periodic literature or its college text books. Of course the one great reason is that our profession as such has not yet risen to a supporting appreciation of authors, and neither authors nor publishers have as yet appeared with sufficient philanthropic enthusiasm to overcome the profession's lack of appreciation. It has been only an occasional overburdened conscience that has dared to tempt the intellectual appetite of our profession with a treatise of any considerable extent, unless he was assured of some monetary return because of his connection with a college where he can make it worth the while of his own students at least to buy his book. Of course there never can be money enough in such ventures to tempt one to undertake the very arduous labor of producing a book from the commercial view; but it does seem as though the profession would sometime come to expect its men of talent to place in permanent and accessible form the results of their successful researches. The present tendency is for book publishers to gather together in a single volume the results of different authorities on different classes of subjects under the editorship of an individual. No one can believe that this will encourage literary achievement by such contributors, for they at once feel sufficiently satisfied with this effort and do not care to further pursue the subject especially to the end of presenting it comprehensively in a book over their own signature. Are we not losing our opportunities for not only enlarging the numerical book output, but in dwarfing our talent which should be developed by exacting more serious and individual contributions? Practically all our books are now made by teachers and with a view to use as text books for instruction. This is a bit of commercialism that the editors of our esteemed *Journal of the Allied Societies* might discuss with at least as much concern as the influence of the "Trade Journal" on professional ideals.

**CAST INLAY PATENTS.** Considerable interest in the question as to the ethical claims of Dr. Taggart as the inventor of the process of casting gold inlays has developed during the past three or four months. The crisis came as a result of Dr. Taggart suing Dr. Boynton of Washington City for damages for using his process and the Jameson Centrifugal Machine. The suit is being strongly contested by Taggart and Boynton, who is supported by a large number in the profession who do not believe Taggart has a just claim to a patent on the process or his machine either. While the suit is undecided as yet the profession has taken up the question on an ethical basis and seems to be strongly divided. We print elsewhere a communication from Dr. Edmund Noyes of Chicago, who probably knows the situation as well as any one can, and he not only stated the true attitude all ethical men would like to take, and which no doubt they would take had the whole matter been presented to them properly in the start. Many are irritated by the suit and feel that we are again to be placed at the mercy of unscrupulous commercial organizations, such as handled the vulcanite license and the crown and bridge patents. We believe that it is not yet too late for Taggart to regain his rights, and in some form the suggestion of Dr. Noyes may materialize. We doubt if the profession will ever stand for another patent royalty, but we believe there are enough men of the higher ethical sensibilities to wholly recompense Dr. Taggart for his financial outlay and to insure him a comfortable income for his very valuable contribution to our technical equipment. There ought not to be any great difficulty in demonstrating the superiority of the Taggart casting apparatus, and there certainly are one thousand dentists in the United States who will agree to purchase one of his outfits providing he will cease litigation and place his machine in the hands of any or all of our regular dealer's hands at any price that will fully compensate him. Some such scheme seems to us much better for all concerned than endless and expensive litigation.

### DR. TAGGART AND THE DENTAL PROFESSION.

*Editor Dental Register:* The editorial in the Dental Review for February entitled "The Tragedy of the Dental Profession" states the case fairly and truthfully and makes pretty clearly apparent the "tragedy" of the situation as it relates to Dr. Taggart. It seems necessary however, if it is possible, to bring more sharply and clearly to the attention and the consciences of the dental profession the duty we owe to Dr. Taggart.

There is no doubt whatever that the dental profession will continue to maintain its opposition to the holding by members in good standing, of "process patents" which require an office license or the payment of royalties upon operations performed, but moral questions are apt to have several aspects and those who have insisted strongly upon the relinquishment of the legal rights and emoluments which an inventor might receive under such a patent have mostly ignored the responsibilities which the profession must assume when it insists upon such an ethical requirement, namely, the obligation to prevent or remedy any instances of gross injustice that may arise by reason of obedience to that ethical rule. If there are any who are insisting upon such compliance by Dr. Taggart and have not yet fully discharged their personal obligation to him it is worth while for them to consider carefully whether they belong to that old sect that received the indignant and scornful rebuke "For they bind heavy burdens and grievous to be born, and lay them on men's shoulders; but they themselves will not move them with one of their fingers." It is to be noted that before taking any steps to enforce his supposed legal rights under his process patent he waited at least eight months after his machines were ready, during which time the dental profession had ample opportunity to show their appreciation of the obligation above refered to and their disposition if they had any, to treat Dr. Taggart with something like justice. Dr. Taggart has spent two years, more or less, of his time, all of his money, and strained his credit to the limit, and whether willingly or unwillingly, he has given it all to the profession, and the profession is in full possession of it all to-day.

Now the solution of this situation is not, chiefly, by organizations and subscriptions, and resolutions by societies or even by appropriations from society treasuries, all those may help some but the only proper and adequate solution is by the individual action of every man who is casting any inlays. Probably there are not a dozen men in the United States who would dare to say positively that they would now be making any cast inlays except for the work and the announcements done by Dr. Taggart. The making of inlays has a money value to every man who is making them and that value he has received from Dr. Taggart and owes to him a just compensation in money for that value received. The whole matter resolves itself, in this aspect of it, into a question of simple, common, every day honesty, exactly the same kind of honesty we all expect our patients to practice toward us and that we wish them to believe that we practice ourselves. There are probably at least one thousand dentists, perhaps there are two thousand) in the state of Illinois who are making cast inlays. If each of these men will pay his debt to Dr. Taggart, in amount having proper relation to the money value of the process to himself, or more properly still by buying Dr. Taggart's machine, the price of which represents Dr. Taggart's judgment as to what he is entitled to receive from those who use his process, it need not be doubted that Dr. Taggart will gladly relinquish all attempts to collect office licenses or royalties. Ingratitude and dishonesty are pretty hard words but it is doubtful if any sophistry, or special pleading, or extenuating circumstances, will enable any man to squirm out from under them if he refuses or neglects to repay his pecuniary obligation to Dr. Taggart. EDMUND NOYES, D.D.S., *Chicago,*

